# Reverse and Forward Electron Flow-Induced H_2_O_2_ Formation Is Decreased in α-Ketoglutarate Dehydrogenase (α-KGDH) Subunit (E2 or E3) Heterozygote Knock Out Animals

**DOI:** 10.3390/antiox11081487

**Published:** 2022-07-29

**Authors:** Gergő Horváth, Gergely Sváb, Tímea Komlódi, Dora Ravasz, Gergely Kacsó, Judit Doczi, Christos Chinopoulos, Attila Ambrus, László Tretter

**Affiliations:** Department of Biochemistry, Semmelweis University, 1094 Budapest, Hungary; horvath.gergo@med.semmelweis-univ.hu (G.H.); svab.gergely@med.semmelweis-univ.hu (G.S.); komlodi.timea@med.semmelweis-univ.hu (T.K.); ravasz.dora@med.semmelweis-univ.hu (D.R.); gergelykacso@gmail.com (G.K.); doczi.judit@med.semmelweis-univ.hu (J.D.); chinopoulos.christos@med.semmelweis-univ.hu (C.C.); ambrus.attila@med.semmelweis-univ.hu (A.A.)

**Keywords:** KGDHc, α-ketoglutarate dehydrogenase complex, OGDHc, oxoglutarate dehydrogenase complex, ischemia-reperfusion, antioxidant systems, DLD, DLST, mitochondria, reactive oxygen species, reverse electron transfer, RET, ROS, cellular respiration, transgenic animal, succinate, α-glycerophosphate

## Abstract

α-ketoglutarate dehydrogenase complex (KGDHc), or 2-oxoglutarate dehydrogenase complex (OGDHc) is a rate-limiting enzyme in the tricarboxylic acid cycle, that has been identified in neurodegenerative diseases such as in Alzheimer’s disease. The aim of the present study was to establish the role of the KGDHc and its subunits in the bioenergetics and reactive oxygen species (ROS) homeostasis of brain mitochondria. To study the bioenergetic profile of KGDHc, genetically modified mouse strains were used having a heterozygous knock out (KO) either in the dihydrolipoyl succinyltransferase (DLST^+/−^) or in the dihydrolipoyl dehydrogenase (DLD^+/−^) subunit. Mitochondrial oxygen consumption, hydrogen peroxide (H_2_O_2_) production, and expression of antioxidant enzymes were measured in isolated mouse brain mitochondria. Here, we demonstrate that the ADP-stimulated respiration of mitochondria was partially arrested in the transgenic animals when utilizing α-ketoglutarate (α-KG or 2-OG) as a fuel substrate. Succinate and α-glycerophosphate (α-GP), however, did not show this effect. The H_2_O_2_ production in mitochondria energized with α-KG was decreased after inhibiting the adenine nucleotide translocase and Complex I (CI) in the transgenic strains compared to the controls. Similarly, the reverse electron transfer (RET)-evoked H_2_O_2_ formation supported by succinate or α-GP were inhibited in mitochondria isolated from the transgenic animals. The decrease of RET-evoked ROS production by DLST^+/−^ or DLD^+/−^ KO-s puts the emphasis of the KGDHc in the pathomechanism of ischemia-reperfusion evoked oxidative stress. Supporting this notion, expression of the antioxidant enzyme glutathione peroxidase was also decreased in the KGDHc transgenic animals suggesting the attenuation of ROS-producing characteristics of KGDHc. These findings confirm the contribution of the KGDHc to the mitochondrial ROS production and in the pathomechanism of ischemia-reperfusion injury.

## 1. Introduction

Alpha-keto (2-oxo) acid dehydrogenase complexes are large multienzyme complexes in the mitochondrial matrix comprising the alpha-ketoglutarate dehydrogenase complex (KGDHc or 2-oxoglutarate dehydrogenase), pyruvate dehydrogenase complex, 2-oxoadipate dehydrogenase complex and branched-chain alpha-keto acid dehydrogenase complex [1,2,3,4,5]. The KGDHc catalyzes a rate-limiting step in the tricarboxylic acid (TCA) cycle converting α-ketoglutarate (α-KG) to succinyl-CoA in an oxidative decarboxylation reaction reducing NAD^+^ to NADH, releasing CO_2_ and utilizing HS-CoA [6,7]. The KGDHc consists of three components/subunits: α-ketoglutarate/2-oxoglutarate dehydrogenase (KGDH/OGDH, E1; EC 1.2.4.2), dihydrolipoyl succinyltransferase (DLST; EC 2.3.1.61), and dihydrolipoyl dehydrogenase (DLD, E3; EC 1.8.1.4) [8,9,10]. The complex is heavily regulated and plays a role in energy coupling [11,12]. α-KG can be a major input of the TCA cycle because α-KG can be produced in the matrix (mouse and human) in 28 reactions, see https://metabolicatlas.org/explore/Mouse-GEM/gem-browser/metabolite/MAM01306m; (accessed on 13 April 2022). Besides producing NADH (substrate for CI in the electron transfer system; ETS) for the terminal oxidation, the KGDHc reaction is also a major source of succinyl-CoA, a substrate for succinyl-CoA ligase (SUCL). The SUCL reaction leads to the formation of ATP or GTP via substrate-level phosphorylation (SLP) which is a major source of mitochondrial ATP in the absence of oxidative phosphorylation [13,14]. In energetically impaired mitochondria, ATP generated by SLP can prevent hydrolysis of the glycolytic ATP via avoiding the reversal of the ADP-ATP transporter, the adenine nucleotide translocase (ANT) [14,15,16]. Mitochondria are the powerhouses of the cell, but like all powerhouses, they also have some less desirable byproducts such as reactive oxygen species (ROS) [17,18]. Although, traditionally ROS production is mainly attributed to the ETS [17,18,19], many observations showed that soluble enzymes in mitochondria can also contribute to ROS formation [20,21,22,23]. Among them, the KGDHc is an important player in mitochondrial ROS production [23,24,25,26] being highly dependent on the NADH/NAD^+^ ratio [23]. 

Taking into account that (*i*) KGDHc is a TCA cycle enzyme with a considerable flux control coefficient [6,27], (*ii*) KGDHc is capable of generating a high amount of ROS [23,25], and (*iii*) mitochondrial ROS generation is often involved in the pathogeneses of neurodegenerative diseases [28], it is not surprising that numerous diseases have been associated with impairments of the KGDHc subunits [29,30,31,32,33,34,35].

In order to assess the role of the KGDHc in brain mitochondrial bioenergetics, oxygen consumption, ROS production, and antioxidant expression were studied. Various genetically modified mouse strains have been used having a heterozygous mutation either in the dihydrolipoyl succinyltransferase (DLST^+/−^) or in dihydrolipoyl dehydrogenase (DLD^+/−^). 

The same genetically modified mouse strains have been used earlier [14,25,36]. Homozygous knock-out animals of either gene are lethal [26,37]. Heterozygotes, however, did not show any phenotypic appearance while being more susceptible to mitochondrial toxins [36,37,38]. This study is fundamentally different from those dealing with mutations of the KGDHc enzyme reporting that impairment of the catalytic function of the enzyme is associated with elevated ROS production [39,40]. Results of this study (*i*) corroborate previous results in vitro and in situ in mitochondria [20,25] that the KGDHc subunit DLD is a major producer of ROS, therefore lack of expression of this subunit results in a direct decrease of ROS production; (*ii*) point out the importance of DLST and DLD subunits of the KGDHc in mitochondrial ROS production; (*iii*) highlight that the lack of DLD subunit also decreases the ROS generation during reverse electron transfer (RET) induced by either succinate, or α-glycerophosphate; and (*iv*) point out that in the absence of a high rate of ROS generation mitochondrial glutathione peroxidase 1 expression is decreased in transgenic mice. Oxygen consumption data presented in this manuscript are consistent with the results published earlier [14,25].

## 2. Materials and Methods

### 2.1. Animals

Heterozygous DLD^+/−^ (DLD^+/−^, C57BL/6) mice and wild type (WT) littermates were obtained from Jackson Laboratory (JAX mice; Jackson Laboratory Repository, Bar Harbor, ME, USA). Mice deficient in the DLST subunit (DLST^+/−^; C57BL/6 and 129SV/EV hybrid) and WT littermates were obtained from Lexicon Pharmaceuticals (The Woodlands, TX, USA). 

WT mice were reproduced by crossing DLD^+/−^ and DLST^+/−^; thus, all WT mice have DLD^+/−^ or DLST^+/−^ progeny. The animals used in our study were of either sex and between 3 and 6 months of age. Mice were housed in a room maintained at 20–22 °C on a 12-h light-dark cycle with food and water available *ad libitum*. Animals were decapitated by a process in accordance with the International Guiding Principles for Biomedical Research Involving Animals and Guidelines for Animal Experiments at Semmelweis University according to the EU Directive “Directive 2010/63/EU of the European Parliament and of the Council of 22 September 2010 on the protection of animals used for scientific purposes”.

### 2.2. Mitochondrial Isolation

Synaptic and non-synaptic mitochondria were isolated from adult mice brain using a discontinuous Percoll gradient, as detailed earlier [41,42]. Brains were immediately removed and homogenized in ice-cold buffer A (in mM: 225 mannitol, 75 sucrose, 5 HEPES, 1 EGTA; pH 7.4) and then centrifuged for 3 min at 1300× *g*. The supernatant was centrifuged for 10 min at 20,000× *g*. The pellet was then suspended in 15% Percoll and layered on a discontinuous gradient consisting of 40% and 23% Percoll layers, which was then centrifuged for 8 min at 30,700× *g*. After resuspension of the lowermost fraction in buffer A, it was centrifuged at 16,600× *g* for 10 min, and then the pellet was resuspended in buffer A and centrifuged again at 6300× *g* for 10 min. After the supernatant was discharged, the pellet was resuspended in buffer B (in mM: 225 mannitol, 75 sucrose, 5 HEPES, pH 7.4) yielding ~30 mg/mL mitochondrial protein concentration. Mitochondrial protein concentration was determined by a modified Biuret assay [43]. Mitochondria were prepared and used within 4 h in each experiment and added to the cuvettes or the O2k-chamber after careful resuspension. Unless otherwise indicated, 0.1 mg/mL mitochondrial protein was applied in the experiments.

### 2.3. Mitochondrial Oxygen Consumption

Mitochondrial oxygen consumption was measured using high-resolution respirometry (Orboros O2k; Oroboros Instruments, Innsbruck, Austria) at 37 °C in 2-mL chambers under continuous stirring [41]. Data were digitally recorded and analyzed. Oxygen concentration was monitored by the polarographic oxygen sensor (POS) and the oxygen flux was calculated as the negative time derivative of the oxygen concentration [44]. POS was calibrated routinely at air saturation and in oxygen-depleted medium. Oxygen consumption was measured in the following standard medium (in mM): 125 KCl, 20 HEPES, 2 KH_2_PO_4_, 0.1 EGTA, 1 MgCl_2_ and 0.025% bovine serum albumine fatty-acid free (BSA), pH 7.4. Mitochondria were energized with α-KG (5 mM), α-glycerophosphate (α-GP; 20 mM), or succinate (5 mM).

### 2.4. Mitochondrial H_2_O_2_ Formation

The rate of H_2_O_2_ generation was determined using the Amplex UltraRed assay [45]. Horseradish peroxidase (5 U per 2 mL), Amplex UltraRed (2 µM), and mitochondria were added to the standard medium. Fluorescence was recorded at 37 °C at 550 nm excitation and 585 nm emission wavelengths in a PTI Deltascan fluorescence spectrophotometer (Photon Technology International, Lawrenceville, NJ, USA). The fluorescence signal was calibrated with known quantities of H_2_O_2_ at the end of each experiment.

### 2.5. Western Blotting

Frozen-thawed mitochondrial pellets were separated by sodium dodecyl sulfate–polyacrylamide gel electrophoresis (SDS-PAGE). Separated proteins were transferred to a methanol-activated polyvinylidene difluoride membrane. Immunoblotting was performed as recommended by the manufacturers of the antibodies. Mouse monoclonal anti-cyclophilin D (cypD; Mitosciences, Eugene, OR, USA), rabbit polyclonals anti-OGDH, anti-DLST, anti-DLD, anti-VDAC1, anti-CypD, anti-GR, anti-GPX1, anti-TRX, and anti-PRX3 (Abcam, Cambridge, UK) primary antibodies were used at concentrations of 1 microg/mL, while rabbit polyclonal anti-manganese superoxide dismutase (MnSOD; Abcam) at 0.2 microg/mL. Immunoreactivity was detected using the appropriate peroxidase-linked secondary antibody (in 1:4000 dilution, donkey anti-mouse or donkey anti-rabbit; Jackson Immunochemicals Europe Ltd., Cambridgeshire, UK) and enhanced chemiluminescence detection reagent (ECL system; Amersham Biosciences GE Healthcare Europe GmbH, Vienna, Austria).

### 2.6. Statistics

Data, in general, are presented as the means ± S.E.M. Normal distribution was tested by the F-probe. Statistical differences were evaluated with ANOVA (SIGMASTAT; Systat Software Inc., San Jose, CA, USA) followed by the Bonferroni’s test for multiple comparison; *p* < 0.05 represents a significant difference.

### 2.7. Materials

All laboratory chemicals were obtained from Sigma Aldrich (St. Louis, MO, US) except for ADP (Merck Group, Darmstadt, Germany), α-GP (Santa Cruz Biotechnology, Dallas, TX, USA), and Amplex UltraRed (ThermoFisher Scientific, Waltham, MA, USA).

## 3. Results

In order to examine the mitochondrial bioenergetics of the KGDHc subunit deficient mice, oxygen consumption, ROS production, and protein expression were monitored in normal versus genetically modified mitochondria.

### 3.1. Oxygen Consumption of Mitochondria Using Various Respiratory Substrates

Mitochondria energized with α-KG isolated from control and transgenic KGDHc heterozygote animals exhibited acceptable coupling and mitochondrial quality (see Table S1). The respiratory control ratio (the rate of respiration in the presence of respiratory substrate and ADP/the rate of respiration in the absence of ADP) is a widely used parameter for the quality control of mitochondrial preparations. Importantly, there were no significant differences between the control and transgenic groups in terms of mitochondrial quality and coupling regardless of the respiratory substrates used.

#### 3.1.1. Respiration of α-Ketoglutarate-Supported Mitochondria

**Figure 1:**
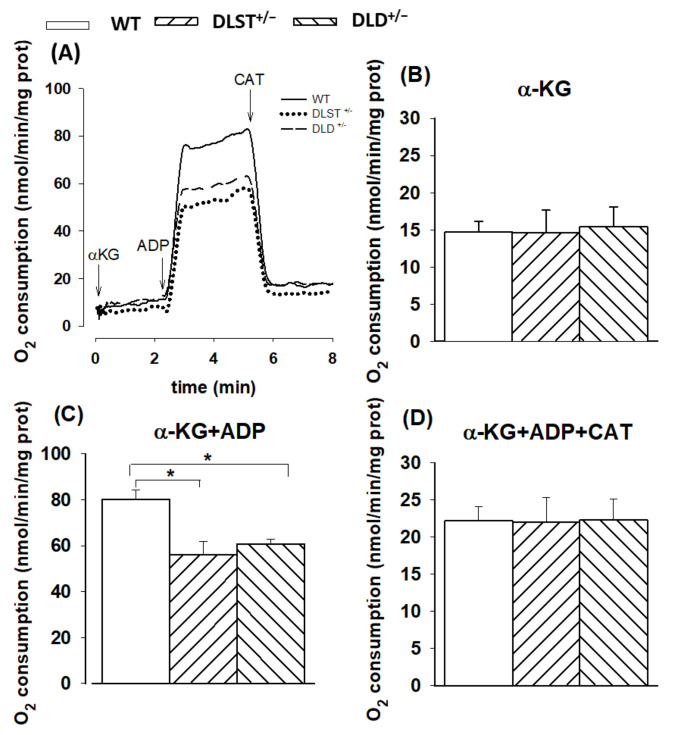
Oxygen consumption of mitochondria isolated from wild-type and KGDHc subunit heterozygote KO mice respiring on α-ketoglutarate (α-KG) in the absence (**B**) or presence (**C**,**D**) of ADP, and after addition of carboxyatractilozide (CAT), (**D**). (**A**) Trace are representatives of experiments with wild-type mice (WT; solid line), dihydrolipoyl succinyltransferase transgenic mice (DLST^+/−^; dotted line), and dihydrolipoyl dehydrogenase transgenic mice (DLD^+/−^; dashed line). Oxygen consumption was monitored by high-resolution respirometry. Mitochondria (0.1 mg/mL) were incubated in a standard medium, as described under Materials and Methods. Afterwards, α-KG (5 mM), ADP (2 mM); (**B**,**C**), and carboxyatractyloside (CAT; 2 µM); (**D**) were given. White bars: WT; bars with left diagonal stripes: DLST^+/−^ mutation; bars with right diagonal stripes: DLD^+/−^ mutation. The results are expressed as the means of oxygen consumption in nmol/min/mg protein ± SEM (*N* = 4–16). Statistically significant differences are indicated by asterisks; * *p* < 0.05.

α-KG is the substrate specific for the KGDHc in mitochondria. In the presence of respiratory substrate (α-KG) but in the absence of ADP, the mitochondrial respiration was similar in the transgenic groups compared to the controls. (Figure 1A). Upon addition of ADP the rate of respiration was elevated, however, in all the transgenic groups a decrease in oxygen consumption was detected compared to the control group. The addition of ADP stimulated the respiration not only via depolarization of the mt-inner membrane but also via direct activation of the KGDHc. After inhibition of the ANT by CAT (Figure 1C), the rate of respiration was decreased in all types of mitochondria. Interestingly, the O_2_ consumption was higher after CAT administration compared to the O_2_ consumption measured in the absence of ADP in mitochondria respiring with α-KG. This phenomenon can be explained by the direct activation of the KGDHc via ADP. 

#### 3.1.2. Respiration of Succinate-Supported Mitochondria

In order to assess whether mutations in various KGDHc subunits affect the oxidation of other substrates independent of the activities of the KGDHc and respiratory Complex I, succinate was also utilized as a respiratory substrate; which is oxidized by Complex II.

**Figure 2:**
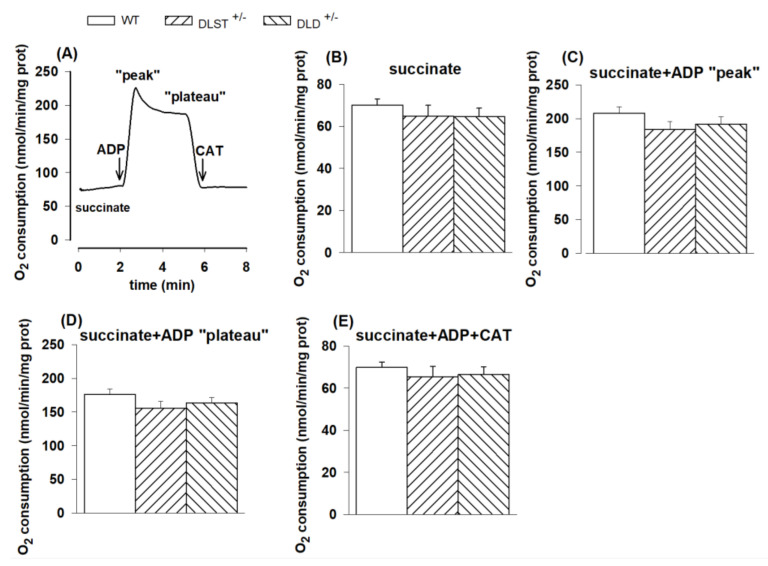
Oxygen consumption of mitochondria isolated from wild-type and KGDHc subunit heterozygote KO mice respiring on succinate in the absence (**B**) or presence (**C**,**D**) of ADP, and after addition of carboxyatractyloside (CAT), (**E**). (**A**) Trace is representative of a single experiment. Succinate (5 mM), ADP (2 mM); (**C**–**E**) and CAT (2 µM); (**E**) were given as indicated. All other conditions and representations are as in Figure 1.

In succinate-energized mitochondria, none of the KGDHc transgenic conditions led to any differences relative to control in the investigated respiratory states (Figure 2A–E). Oxygen consumption in succinate-supported mitochondria in the presence of ADP exhibited a sharp peak (Figure 2A), which was followed by a decline and plateau. These phenomena were potentially attributed to the accumulation of oxaloacetate [46,47], the physiologic inhibitor of succinate dehydrogenase.

#### 3.1.3. Respiration of α-Glycerophosphate (α-GP) Supported Mitochondria

**Figure 3:**
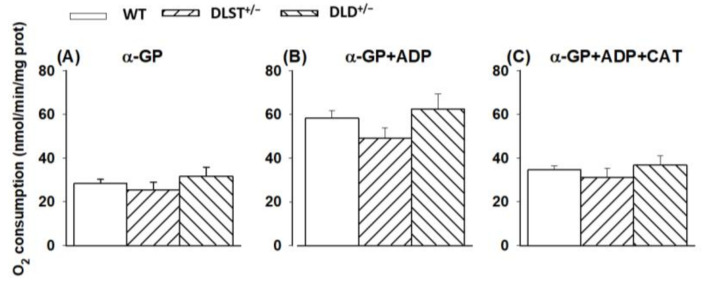
Oxygen consumption of mitochondria isolated from wild-type and KGDHc subunit heterozygote KO mice respiring on α-glycerophosphate in the absence (**A**) or presence (**B**,**C**) of ADP, and after the addition of carboxyatractyloside (CAT), (**C**). α-GP (20 mM), ADP (2 mM); (**B**,**C**) and CAT (2 µM); (**C**) were given as indicated. All other conditions and representations are as in Figure 1.

Similar to succinate, α-GP also donates electrons to coenzyme Q in the ETS without using Complex I during the forward electron transfer. α-GP, unlike succinate, does not enter the mitochondrial matrix, it is oxidized on the outer surface of the mitochondrial inner membrane by α-glycerophosphate dehydrogenase (α-GPDH) [48,49,50]. Furthermore, the α-GPDH shuttle can mediate the oxidation of cytosolic (glycolytic) NADH into the mitochondria. Brain mitochondria possess a high α-GPDH activity [50,51], therefore, using this substrate could serve as a good control for the effects of the KGDHc mutations on the ETS. In line with the results on succinate oxidation, there were no significant differences between the groups of transgenic animals compared to the control under the conditions applied (Figure 3).

### 3.2. Mitochondrial H_2_O_2_ Production

Considering that the KGDHc subunits are capable of producing ROS, even in their liberated forms [52], the aim of the experiments was to determine the effects of partially missing subunits on mitochondrial ROS production. The formation of H_2_O_2_, the most stable form of ROS, was studied according to the following experimental setup: (*i*) mitochondria were energized with a single substrate (α-KG, succinate, or α-GP); (*ii*) ADP was added to generate a high rate of respiration via decreasing the Δ*Ψ*_m_ and supplying substrate for the ATP synthase; (*iii*) an ANT inhibitor was given to re-establish the hyperpolarized Δ*Ψ*_m_ and prevent the transport of extramitochondrial ADP to the matrix; (*iv*) rotenone, an inhibitor of the Complex I, was administered to inhibit the reverse electron transfer (RET) evoked by either succinate or α-GP; and (*v*) antimycin A was added to block electron transport at Complex III.

#### 3.2.1. H_2_O_2_ Production of α-Ketoglutarate-Supported Mitochondria

**Figure 4:**
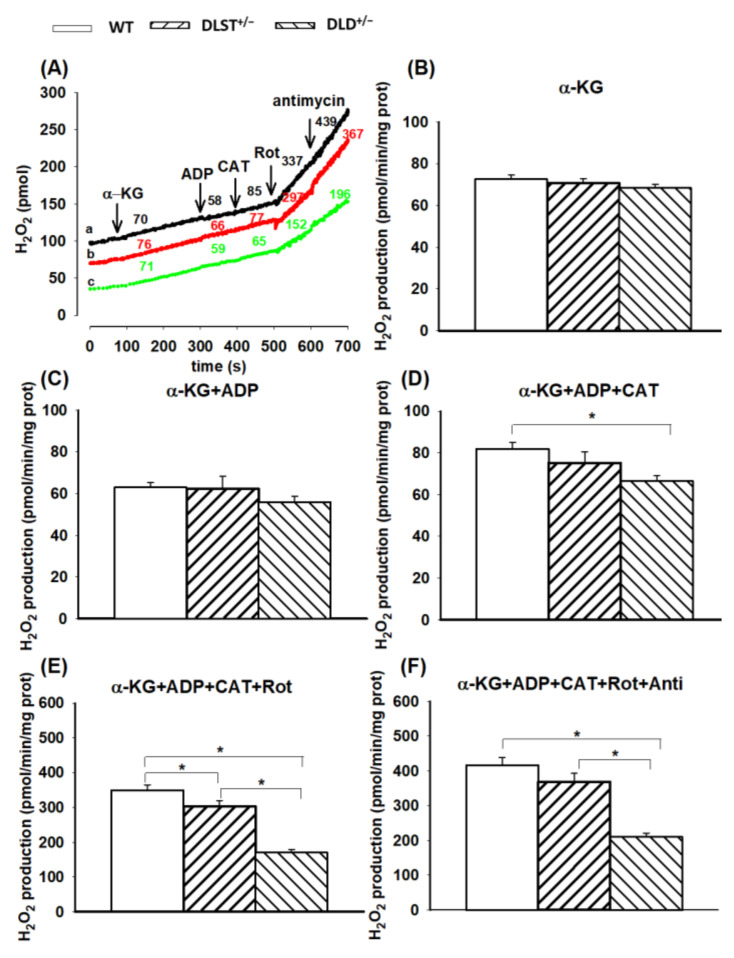
Hydrogen peroxide production in wild-type and KGDHc-subunit-KO mitochondria respiring on α-ketoglutarate. H_2_O_2_ production was measured with the Amplex UltraRed assay as described under Materials and Methods. (**A**) Traces represent single independent experiments and are offset for clarity. Trace *a* (black): WT, trace *b* (red): DLST^+/−^, trace *c* (green): DLD^+/−^. Mitochondria (0.1 mg/mL) were incubated in the standard medium which was followed by the addition of α-KG (5 mM), ADP (2 mM), CAT (2 µM), rotenone (Rot, 250 nM), and antimycin A (1 µM), as indicated. Numbers on the traces represent rates of H_2_O_2_ generation expressed in pmol/min/mg protein. H_2_O_2_ production was measured in the presence of α-KG (**B**) and after adding ADP (**C**–**F**), CAT (**D**–**F**), Rot (**E**,**F**) and antimycin A (**F**). Results are expressed as means of rates of H_2_O_2_ production in pmol/min/mg protein ± S.E.M. (*N* = 4–14). All other representations are as in Figure 1**.** Statistically, significant differences are indicated by asterisks; * *p* < 0.05.

In α-KG-supported mitochondria (with no other additions), the following tendency in ROS production was found: WT > DLST^+/−^ > DLD^+/−^, but there was no statistically significant difference among the WT and transgenic groups (Figure 4B). As was expected, in the presence of ADP, there was a slight decrease in H_2_O_2_ production in every group, however, significant differences could still not be detected in the transgenic groups compared to the control (Figure 4C). Inhibition of ANT by CAT elevated the rate of H_2_O_2_ formation owing to hyperpolarization of Δ*Ψ*_m_ (Figure 4D). In the DLD^+/−^ transgenic groups, H_2_O_2_ formation was significantly decreased relative to the control after CAT addition. Adding CI inhibitor rotenone, H_2_O_2_ production was accelerated in every group, and differences between the control and the transgenic groups became significant (Figure 4E). Inhibition of Complex III by antimycin A added after rotenone only slightly stimulated the H_2_O_2_ formation, while the H_2_O_2_ production was again smaller relative to control in all the transgenic groups in the presence of antimycin A (Figure 4F). It is worth mentioning that the decrease in the H_2_O_2_ production was the smallest in the DLST^+/−^ mitochondria. Therefore, most of the decrease in H_2_O_2_ production could be ascribed to the lost allele of DLD.

#### 3.2.2. H_2_O_2_ Production of Succinate-Supported Mitochondria

**Figure 5:**
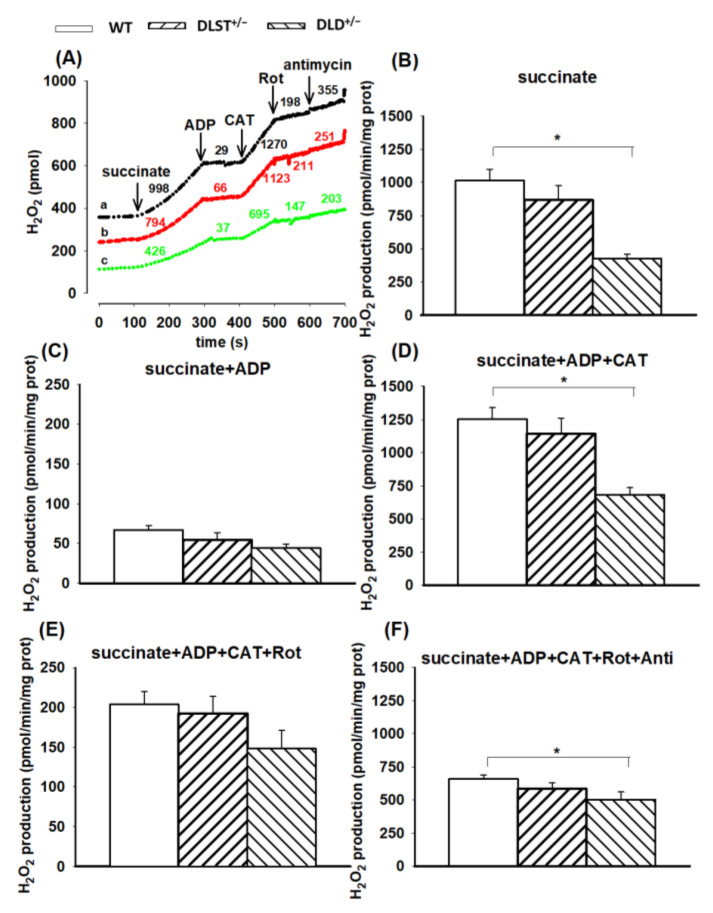
Hydrogen peroxide production in the wild-type and KGDHc-subunit-KO mitochondria respiring on succinate. H_2_O_2_ production was measured with the Amplex UltraRed assay as described under Materials and Methods. (**A**) Traces represent single independent experiments and are offset for clarity. Trace *a* (black): wild-type (WT), trace *b* (red): DLST^+/−^, trace *c* (green): DLD^+/−^. Mitochondria (0.1 mg/mL) were incubated in the standard medium which was followed by the addition of succinate (5 mM), ADP (2 mM), carboxyatractilozide (CAT, 2 µM), rotenone (Rot, 250 nM), and antimycin A (2 µM) as indicated. Numbers on the traces represent H_2_O_2_ production expressed in pmol/min/mg protein. H_2_O_2_ production was measured in the presence of succinate (**B**), after adding ADP (**C**–**F**), CAT (**D**–**F**), rotenone (**E**,**F**) and antimycin A (**F**). All other conditions and representations are as in Figure 1. Statistically significant differences are indicated by asterisks; * *p* < 0.05.

Succinate-evoked ROS formation has several components. Succinate dehydrogenase (SDH) is a flavoenzyme with its own ROS-forming ability [53,54,55]. Under selected conditions (e.g., in the absence of ADP), electrons from succinate can flow backwards passing through Complex I in the reverse direction and reduce NAD^+^ to NADH + H^+^. This pathway is referred to as the reverse electron transfer (RET) [56,57] where the rate of ROS formation is an order of magnitude higher than in the normal flow from Complex II towards Complexes III and IV [45,58,59,60]. As can be seen in Figure 5B, the wild-type mitochondria exhibited the highest rate of H_2_O_2_ formation in the presence of succinate. In the KO groups, ROS formation (except the DLST^+/−^ group) was significantly less than that of the control. The addition of ADP abolished the conditions necessary for RET [60], therefore, the forward flow of electrons evoked a less intensive H_2_O_2_ formation (Figure 5C). Under these conditions, there were no significant differences detected among the individual mitochondrial groups. Inhibition of ADP entry into the mitochondria, applying the ANT inhibitor CAT, reestablished the conditions favorable for RET, and again, significant differences in ROS formation were detected among the WT and transgenic mitochondria (Figure 5D). After blocking the ANT, mitochondria were treated with the Complex I inhibitor rotenone (Figure 5E). Contrary to that found with α-KG, which generates the Complex I substrate NADH, the addition of rotenone to succinate-supported mitochondria decreased the rate of H_2_O_2_ production, which was attributed to the inhibition of RET. Similar to those results observed in the presence of ADP, the significant differences among the individual groups also disappeared. The H_2_O_2_ production in the wild-type mitochondria after adding rotenone decreased from 1253.8 ± 318.8 to 203.6 ± 30.6 pmol/min/mg protein. A similar decrease was detected in the DLD^+/−^ transgenic mice (from 682.2 ± 54.2 to 148.1 ± 23.0 pmol/min/mg protein) after rotenone addition. Administration of antimycin A to rotenone-treated mitochondria stimulated the H_2_O_2_ production, indicating that inhibition at the CIII increased the electron leak from CoQ and the FAD prosthetic group of SDH (Figure 5F).

#### 3.2.3. H_2_O_2_ Production by α-Glycerophosphate-Supported Mitochondria

**Figure 6:**
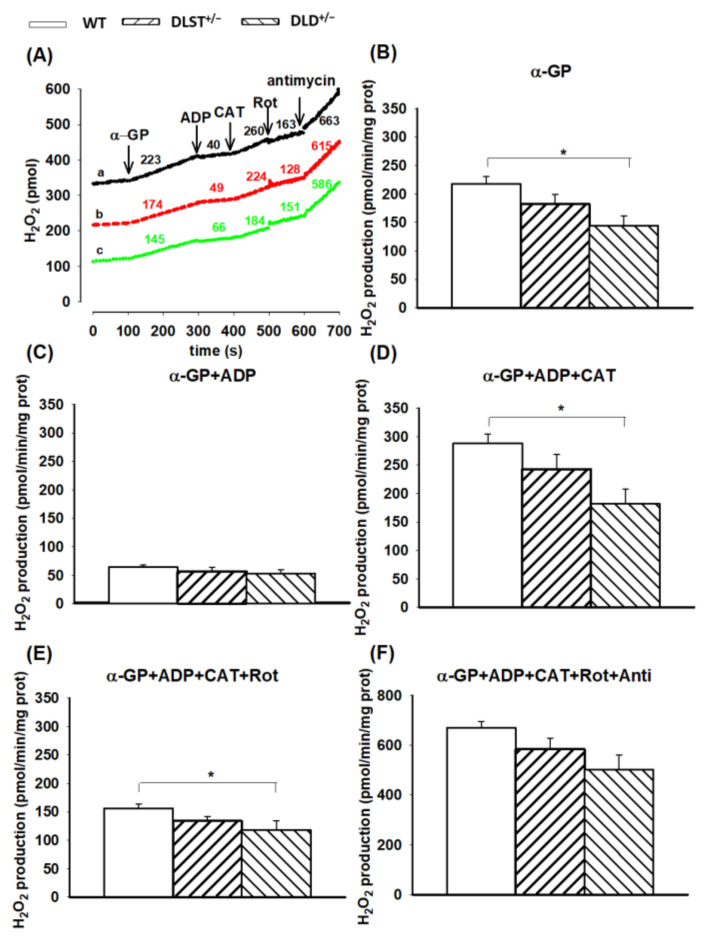
Hydrogen peroxide production in the wild-type and KGDHc-subunit-KO mitochondria respiring on α-glycerophosphate. H_2_O_2_ production was measured with the Amplex UltraRed assay as described under Materials and Methods. (**A**) Traces represent a single independent experiment and are offset for clarity. Trace *a* (black): wild-type (WT), trace *b* (red): DLST^+/−^, trace *c* (green): DLD^+/−^. Mitochondria (0.1 mg/mL) were incubated in the standard medium. Afterwards, α-GP (20 mM), ADP (2 mM), and carboxyatractyloside (CAT; 2 µM), rotenone (Rot; 250 nM), and antimycin A (2 µM) were given as indicated. Numbers on the traces represent H_2_O_2_ production expressed in pmol/min/mg protein. H_2_O_2_ production was measured in the presence of α-GP (**B**), after adding ADP (**C**–**F**), CAT (**D**–**F**), rotenone (**E**,**F**) and antimycin A (**F**). All other conditions and representations are as in Figure 1. Statistically significant differences are indicated by asterisks; * *p* < 0.05.

We reported earlier that α-GP is capable of supporting RET in brain mitochondria [61,62] which is strongly dependent on the concentration of the substrate and the exogenous Ca^2+^. Here, the H_2_O_2_ production using α-GP and succinate showed a similar pattern in the absence of ADP (Figure 5 and Figure 6). In the α-GP respiring mitochondria, however, significant differences were found only between the WT and DLD^+/−^ heterozygotes in the absence of ADP and upon inhibition of the ANT and CI (Figure 6). 

### 3.3. Protein Expression of the KGDHc Subunits in the Wild-Type and Transgenic Animals

**Figure 7:**
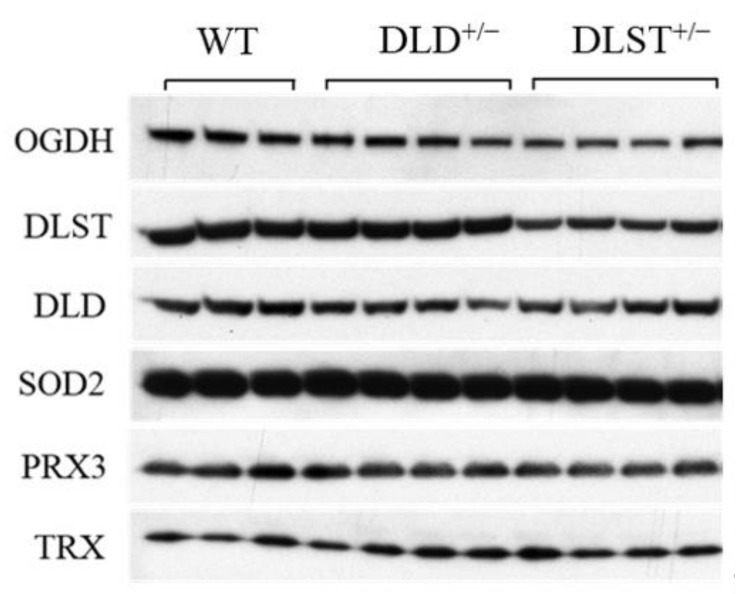
Immunoreactivities of alpha-ketoglutarate dehydrogenase (KGDH/OGDH), dihydrolipoyl succinyltransferase (DLST), dihydrolipoyl dehydrogenase (DLD), superoxide dismutase 2 (SOD2), peroxiredoxin 3 (PRX3), and thioredoxin (TRX) in mitochondria isolated from brains of wild-type (WT), DLST^+/−^, and DLD^+/−^ transgenic mice.

**Figure 8:**
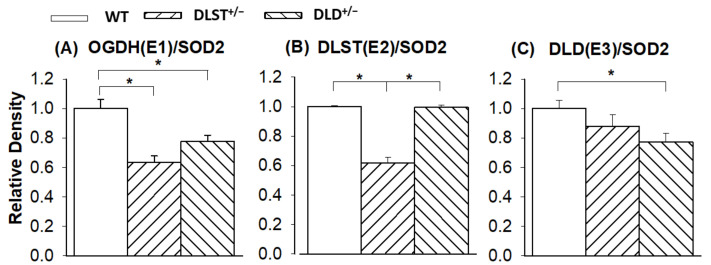
Western blot analysis and relative density changes for the KGDHc subunits, normalized for superoxide dismutase 2 (SOD2) protein expression, in brain mitochondria isolated from the wild-type and KGDHc-subunit-KO mice. (**A**) alpha-ketoglutarate/2-oxoglutarate (KGDH/OGDH) subunit; (**B**) dihydrolipoyl succinyltransferase (DLST) subunit; (**C**) dihydrolipoyl dehydrogenase (DLD) subunit. White bars: wild-type (WT); bars with left diagonal stripes: dihydrolipoyl succinyltransferase mutation (DLST^+/−^); bars with right diagonal stripes: dihydrolipoyl dehydrogenase mutation (DLD^+/−^). Results are expressed as means of the relative densities ± S.E.M. (*N* = 3–4). Statistically significant differences are indicated by asterisks; * *p* < 0.05.

Next, we investigated how the genetic manipulations affected the protein expression of the KGDHc subunits in the mitochondria isolated from the transgenic animals compared to the controls (Figure 7 and Figure 8). In Figure 8 KGDHc subunit expression levels were normalized to the expression of SOD2. In all of the transgenic constructs, the E1 subunit (OGDH) expression was downregulated compared to the wild type (Figure 8A). As was expected, the expression level of the E2 (DLST) subunit was lower in the DLST^+/−^ mitochondria, relative to control (Figure 8B). As it was expected, the expression of the E3 (DLD) subunit decreased in the DLD^+/−^ animals (Figure 8C).

### 3.4. The Expression and Activities of Enzymes Participating in the Antioxidant System of Mitochondria

**Figure 9:**
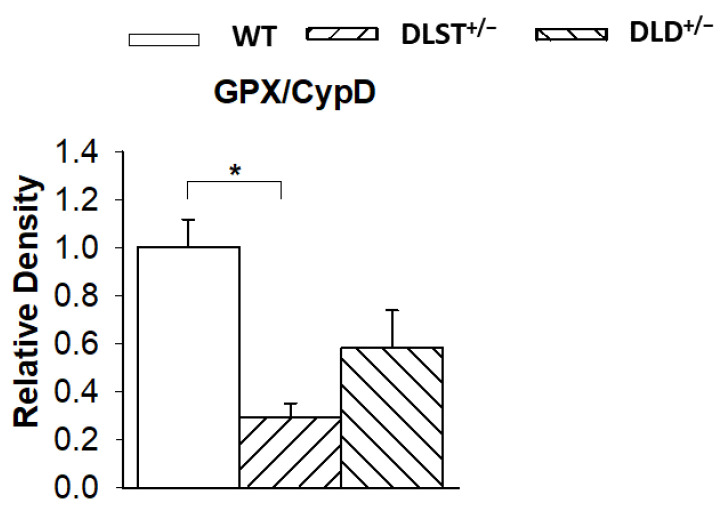
Western blot analysis and relative density changes for glutathione peroxidase (GPX), normalized for cyclophilin D (CypD) protein expression, in brain mitochondria isolated from the wild-type and KGDHc-subunit-deficient mice. White bars: wild-type (WT); bars with left diagonal stripes: dihydrolipoyl succinyltransferase mutation (DLST^+/−^); bars with right diagonal stripes: dihydrolipoyl dehydrogenase mutation (DLD^+/−^). Results are expressed as means of the relative densities ± S.E.M. (*N* = 3–4). Statistically significant differences are indicated by asterisks; * *p* < 0.05.

It was expected that a decrease of ROS production in the subunit deficient KGDHc downregulates the expression of selected mitochondrial antioxidant enzymes. In fact, the thiol-disulfide exchange reactions tightly link the lipoyl residues of the DLST enzyme to glutathione, thioredoxin, and the peroxidases using these reductants to scavenge H_2_O_2_ (reviewed by [26]). Therefore, the activities of glutathione peroxidase (GPX) and glutathione reductase (GR), as well as the expression levels of thioredoxin and peroxiredoxin were all assessed in the transgenic animals and compared to the respective controls. The GPX has a mitochondrial and a cytosolic form [63,64], however, the western blot assays were carried out with mitochondrial lysates, hence detecting only the mitochondrial isoform (Figure 9). In all transgenic groups, there was a notable decrease in the GPX expression (normalized for CypD) compared to the control group suggesting the downregulation of GPX when less ROS was produced in the transgenic animals. Similarly, GR activity normalized for CypD showed a decrease in the transgenic groups compared to the WT mitochondria (Figure S1A). However, no significant differences were detected in the thioredoxin (TRX)/SOD and peroxiredoxin (PRX)/SOD expression ratios normalized for SOD (Figure S1B,C).

## 4. Discussion

The KGDHc catalyzes a rate-limiting step in the TCA cycle, a crossroad of many metabolic processes [6]. Therefore, it is not surprising that reduced activity of the enzyme has even been identified in many age-related neurodegenerative diseases such as Alzheimer’s disease [29,30,31]. The aim of the present study was to reveal the role of the KGDHc and its components/subunits in mitochondrial bioenergetics and redox homeostasis. In particular, ROS generation in brain mitochondria isolated from wild-type and DLD^+/−^ and DLST^+/−^ transgenic mice was investigated. Based on our results, we could conclude that the α-KG-dependent O_2_ consumption was affected in the transgenic animals. It was not the case though with succinate and α-GP, which do not demand a fully functional KGDHc for their oxidation. Importantly, in all the KGDHc-subunit-deficient mitochondria the H_2_O_2_ production was decreased relative to the respective controls not only in the α-KG energized mitochondria but also in mitochondria respiring with succinate and α-GP. The experimental setup we used is entirely different from that used in studies dealing with mutations in the genes responsible for the expression of KGDHc subunits [39,40]. The common feature of the mutations is that they alter both the catalytic and the ROS forming properties of the subunits. Depending upon the type of mutations ROS production can be higher or lower than that of the controls. 

### 4.1. Substrate-Dependent Alterations in Mitochondrial Oxygen Consumption

O_2_ consumption is a sensitive parameter for the mitochondrial bioenergetic status, hence, mitochondrial respiration might well reflect on the impairments or enzyme mutations in the ETS or TCA cycle. Specific respiratory pathways get activated in isolated mitochondria (or other types of mitochondrial preparations, such as permeabilized cells) when applying fuel substrates such as α-KG, succinate, or α-GP in the presence or absence of ADP and specific inhibitors of the ETS [65] respiring on succinate and α-GP. Throughout the present study, the O_2_ consumption was monitored in the investigated mitochondria by applying various respiratory substrates.

#### 4.1.1. Mitochondrial O_2_ Consumption in α-KG-Supported Mitochondria

In accord with earlier reports [14,25,36], O_2_ consumption was not lower in mitochondria isolated from either DLD^+/−^ or DLST^+/−^ transgenic mice in the absence of ADP (Figure 1). This might be explained by the fact that there are endogenous respiratory substrates present which could at least partially compensate for the decrease in the KGDHc activity in the DLST^+/−^ and DLD^+/−^ mice. In the presence of ADP, respiration proved to be highly stimulated. This effect could be attributed to the ADP-dependent depolarization of Δ*Ψ*_m_, to the stimulatory effect of ADP on the KGDHc [66], and to ADP-dependent disinhibition of KGDHC by succinyl-CoA due to the participation of ADP in the succinyl-thiokinase reaction. Apart from the disinhibition, the succinyl-CoA discharge in the presence of ADP also releases the KGDHC substrate CoA for enzyme turnover [24]. Owing to the ADP-induced decrease in Δ*Ψ*_m,_ the proton pump activities of the respiratory complexes became stimulated, which led to an augmented O_2_ consumption. As was depicted in Figure 1C, the O_2_ consumption rate significantly decreased in all the KGDHc-subunit-deficient mitochondria compared to the controls after ADP addition. Nevertheless, there were no significant differences in the RCR values compared to WT, suggesting that these transgenic mitochondria could still produce ATP, as was previously reported by Kiss et al. [14]. The effect of ADP was suspended with the ANT inhibitor CAT, leading to a strong decline in O_2_ consumption (Figure 1D). This phenomenon indicates the presence of a normal ADP- and likely Δ*Ψ*_m_ -dependent regulation of the substrate oxidation, not only in the WT, but also in the transgenic mitochondria.

#### 4.1.2. Mitochondrial O_2_ Consumption in Mitochondria Supported by Succinate and α-Glycerophosphate

Succinate and α-glycerophosphate are two mitochondrial energy donor substrates not having any direct connection with α-KG or KGDHc, therefore it was not surprising that their oxidation was not different in the KO animals. 

### 4.2. H_2_O_2_ Production in Mitochondria Using Various Respiratory Substrates

The mitochondrion is a major source of ROS under selected pathological conditions [17,18,19]. A growing body of evidence reveals that matrix dehydrogenases, such as the KGDHc, are capable of producing ROS under specific conditions when the NADH/NAD^+^ ratio is rather high [21,23,24,67,68]. In this study, one of the research objectives was to explore how deletion of the E2 or E3 subunit of the KGDHc affects the ROS-producing sites in brain mitochondria. For this to be accomplished, we utilized various respiratory substrates such as α-KG, succinate, and α-GP, and specific inhibitors of the ETS.

#### 4.2.1. H_2_O_2_ Production in Mitochondria Respiring on α-Ketoglutarate

In agreement with earlier studies, H_2_O_2_ production was decelerated, relative to the respective controls, in KGDHc- E3 subunit-deficient mitochondria with α-KG as respiratory substrate [25] (Figure 4). As was expected, the addition of ADP lowered the ROS production rate likely due to the decrease (depolarization) of Δ*Ψ*_m,_ and NADH/NAD^+^ ratio [23,25,59,60] (Figure 4C). CAT, an inhibitor of the ANT, reestablished the high Δ*Ψ*_m_ and NADH/NAD^+^ ratio and enhanced the H_2_O_2_ production (Figure 4D). In the presence of CAT, the H_2_O_2_ production significantly decreased in the DLD^+/−^ transgenic mitochondria compared to the controls. Rotenone blocked the electron flow at Complex I and further elevated the H_2_O_2_ formation (Figure 4E). The reduced rate of H_2_O_2_ production in the transgenic mitochondria refers to the determining roles of the KGDHc subunits in mitochondrial H_2_O_2_ production. Antimycin A, acting at Complex III, exerted only a minimal stimulatory effect on the H_2_O_2_ production, indicating that in the presence of rotenone only a relatively small percentage of the electrons could reach the ROS-forming site(s) of the Complex III (Figure 4F). 

Considering the multienzyme nature of the KGDHc, this finding is in agreement with a previous reports [20,22,52] that all the three catalytic subunits of the KGDHc participate in the ROS-producing activity thus it is also a possibility that in the absence of DLST the production of free radicals on the OGDH subunit could be accelerated. 

#### 4.2.2. H_2_O_2_ Production in Mitochondria Respiring on Succinate and α-Glycerophosphate

In the absence of ADP, succinate-supported mitochondria exhibited an extremely high rate of ROS production which can be attributed to the reverse electron transfer (RET) [45,57,58]. During RET, electrons from Complex II flow back towards Complex I and reduce NAD^+^ to NADH at high Δ*Ψ*_m_. Similar to that detected in the α-KG-supported mitochondria, the H_2_O_2_ production rate decreased in the transgenic mitochondria, which was the most pronounced in the DLD^+/−^ mitochondria, indicating that under this condition indeed the DLD is the most important player in the course of ROS production. In the presence of ADP, there was a dramatic decrease observed in the rate of H_2_O_2_ formation, which could be attributed to the ADP-induced depolarization of Δ*Ψ*_m_ and thus the abolishment of RET [58,59] (Figure 6C). Importantly, the decrease in the H_2_O_2_ production rate upon ADP addition was more pronounced in succinate-supported mitochondria as compared to the ones respiring on α-KG. This could be explained by the fact that the RET is more sensitive to Δ*Ψ*_m_. In the presence of CAT, the inhibition of ANT hyperpolarizes the mitochondrial inner membrane, hence the conditions get favorable for the RET, and mitochondrial ROS production gets stimulated (Figure 4D). Under this condition, in the DLST^+/−^ and DLD^+/−^ KO animals, the rate of ROS production decreased compared to the control. ROS production was the slowest for DLD^+/−^ indicating again that the DLD subunit is the most important ROS producer when the RET is active (Figure 5B,D). Similar observations were made with the mitochondria energized by α-GP (Figure 6B,D), where all of the NADH formed in the mitochondrial matrix originated from the RET [61]. Inhibition of the ANT brought the ROS formation back to the level detected before the addition of the ADP, consequently, the electrons must at least partially flow in the reverse direction. Administration of rotenone ceased RET and decreased the ROS production indicating that RET was the major source of ROS in the presence of succinate. 

### 4.3. Protein Expression Levels

Surprisingly, in our study the DLST^+/−^ heterozygote KO-s decreased not only the protein expression level of DLST, but also that of the OGDH subunit (Figure 8). Contrary to our results, Yang et al. [36] demonstrated only a decreased expression of the DLST subunit in the DLST^+/−^ animals. This difference might be explained by the different reference proteins and different antibodies used for normalization in these studies. 

Cellular and mitochondrial antioxidant enzymes play key roles in the regulation of redox homeostasis. Under physiological conditions, antioxidants keep the ROS level low preventing the cell or mitochondria from a burst of oxidative stress. Thus, their expression levels reflect a potential redox imbalance and let us conclude on the rate of ROS formation. Therefore, we investigated the protein levels of selected antioxidant enzymes such as GPX, GR, TRX, and PRX3 in mitochondria isolated from the wild-type and KGDHc subunit deficient mice. Surprisingly, only the protein levels of GPX and GR, but not of TRX and PRX, were reduced in the KGDHc subunit deficient transgenic animals (Figure 9 and Figure S1). Conversely, Yang et al. [36] have revealed no difference in the protein levels of the mitochondrial antioxidant enzymes (GPX, GR, MnSOD) in the DLST^+/−^ heterozygotes compared to the controls. These discrepancies might again be explained by the different reference proteins used for normalization in these studies. It is noteworthy that reduced protein levels of GPX and GR support the data on the decreased rate of ROS formation in the KGDHc-subunit-deficient transgenic groups. The lowered expression levels of glutathione-dependent antioxidant enzymes can be considered as an adaptation to the lower rate of ROS production in the mitochondria.

### 4.4. Relevance of Our Data in Ischemia-Reperfusion Injury

**Figure 10:**
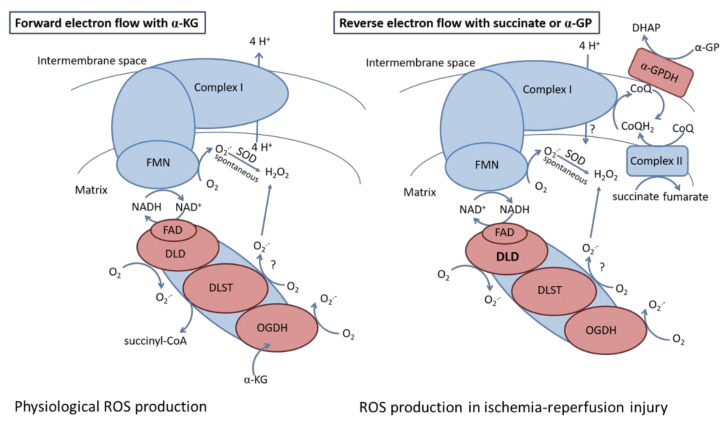
The role of KGDH in ROS production observed during forward and reverse electron transfer in ischemia-reperfusion injury. Abbreviations: α-GP: α-glycerophosphate; α-GPDH: α-glycerophosphate dehydrogenase; α-KG: α-ketoglutarate; CoQ: oxidized coenzyme Q; CoQH_2_: reduced coenzyme Q; DHAP: dihidroxiaceton-phosphate; DLD: dihidrolypoil dehydrogenase; DLST: dihydrolipoaminde succinyltransferase; FAD: flavin adenine dinucleotide; FMN: flavin adenine mononucleotide; OGDH: oxoglutarate dehydrogenase; SOD: superoxide dismutase.

Ischemia-reperfusion injury is associated with disturbed mitochondrial metabolism. During ischemia, succinate accumulates owing to the reversal of succinate dehydrogenase [69] or due to the canonical operation of the Krebs cycle partially supported by aminotransferase anaplerosis [70]. Reperfusion is associated with high ROS production supported by reverse electron transfer induced via the oxidation of the accumulated succinate. As it has been reported by others [71,72,73], the FMN subunit of Complex I might be responsible for most of the ROS produced during RET. Hereby we demonstrate that heterozygous KO of KGDHc subunits decreased the succinate- and α-GP- evoked H_2_O_2_ production under conditions favoring RET. Our data indicate that heterozygous KO of the DLD subunit resulted in a 58% decline in ROS production with succinate and 34% with α-GP. Therefore, these results suggest that the DLD subunit can contribute to the RET-evoked ROS production (Figure 10). Our data confirm the results of Starkov et al. 2004 emphasizing the role of NADH-dependent KGDHc in contribution to RET-evoked ROS formation [25]. To the best of our knowledge, we have shown for the first time that α-GP-induced RET, which can occur in the brain having high α-GPDH activity, was also sensitive to heterozygous KO of DLD subunit. This might have an importance during ischemia when α-GP can accumulate as proven by Ben-Yoseph et al., 1993 [74] and Nguyen et al. 2007 [75] in ischemic in rat brain. 

## 5. Conclusions

Our results highlight the importance of the KGDHc subunits in mitochondrial ROS formation. Decreased ROS production was detected in DLST^+/−^ or DLD^+/−^ KO animals not only in the forward mode of the enzyme (in the presence of α-KG as substrate) but also under the circumstances of reverse electron flow (when NADH is formed by the reverse flow of electrons via CoQ to Complex I). This latter finding highlights the importance of KGDHc subunits in reperfusion-mediated oxidative tissue damage. In the transgenic animals investigated, not only the ROS production was decelerated, but the expression levels of the studied antioxidant enzymes were also attenuated.

## Data Availability

Not applicable.

## Supplementary Materials

The following supporting information can be downloaded at: https://www.mdpi.com/article/10.3390/antiox11081487/s1, Figure S1: Western blot analysis and relative density changes for protein expression in mitochondria isolated from wild-type and KGDHc-subunit-deficient mice.; Table S1: (A): Respiratory control ratio (RCR) in mitochondria isolated from wild-type (WT) and KGDHc-subunit-deficient mice. (B): *P*-*L* control efficiency (OXPHOS coupling efficiency) in mitochondria isolated from wild-type (WT) and KGDHc-subunit-deficient mice.
