# Supplementary material for: Reverse and Forward Electron Flow-Induced H2O2 Formation Is Decreased in α-Ketoglutarate Dehydrogenase (α-KGDH) Subunit (E2 or E3) Heterozygote Knock Out Animals
Source: Antioxidants (Basel). 2022 Jul 29;11(8):1487. doi: 10.3390/antiox11081487 (PMC9404749; doi:10.3390/antiox11081487)
Supplement: Supplementary file 1 [file antioxidants-11-01487-s001.zip › antioxidants-1797695-supplementary.pdf]

Supplementary materials

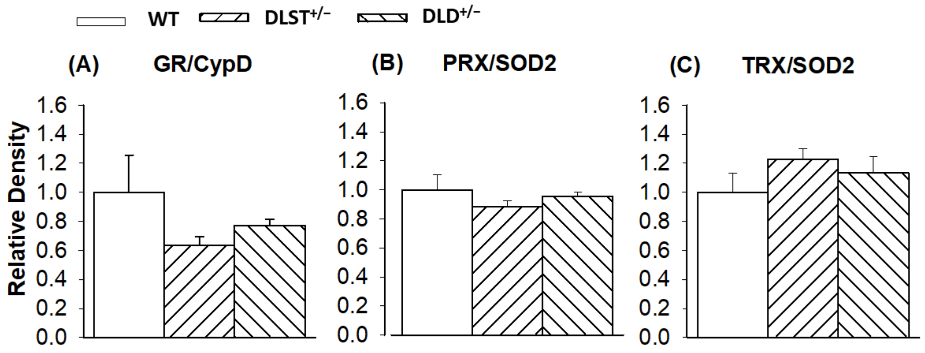

Figure S1. Western blot analysis and relative density changes for protein expression in mitochondria isolated from wild-type and KGDHc-subunit-deficient mice. (A) Glutathione reductase (GR) normalized for cyclophilin D (CypD); (B) peroxiredoxin (PRX) normalized for superoxide dismutase 2 (SOD2); (C) thioredoxin (TRX) normalized for SOD2. White bars: wild-type (WT); bars with left diagonal stripes: dihydrolipoyl succinyltransferase mutation ($DLST^{+/-}$); bars with right diagonal stripes: dihydrolipoyl dehydrogenase mutation ($DLD^{+/-}$). The results are expressed as means of the relative densities±S.E.M. (*N*=3-4).

	WT	DLST$^{+/-}$	DLD$^{+/-}$
α-KG	5.99±0.50	4.48±0.60	4.26±0.65
succinate	2.97±0.07	2.87±0.08	2.92±0.07
α-GP	2.08±0.07	1.99±0.09	1.97±0.10

Table S1A. Respiratory control ratio (RCR) in mitochondria isolated from wild-type (WT), dihydrolipoyl succinyltransferase DLST$^{+/-}$, and dihydrolipoyl dehydrogenase DLD$^{+/-}$ transgenic mice. α-ketoglutarate (α-KG; 5 mM), succinate (5 mM), or α-glycerophospate (α-GP; 20 mM) were used as respiratory fuel substrates. RCR was calculated as the ratio of the O_2 consumption rates measured in the presence and absence of ADP (2 mM). Original traces and data are shown in Figures 1-3. The results are expressed as mean±S.E.M. (*N*=4-16).

	WT	DLST$^{+/-}$	DLD$^{+/-}$
α-KG	0.81±0.02	0.75±0.03	0.75±0.04
succinate	0.66±0.01	0.65±0.01	0.66±0.01
α-GP	0.51±0.01	0.49±0.02	0.49±0.03

Table S1B. *P-L* control efficiency (OXPHOS coupling efficiency) in mitochondria isolated from wild-type (WT), dihydrolipoyl succinyltransferase DLST$^{+/-}$, and dihydrolipoyl dehydrogenase DLD$^{+/-}$ transgenic mice. α-ketoglutarate (α-KG; 5 mM), succinate (5 mM), or α-glycerophospate (α-GP; 20 mM) were used as respiratory fuel substrates. *P-L* (OXPHOS) control efficiency was calculated as [1-(O_2 consumption measured without ADP/O_2 consumption measured with ADP)]. 1 refers to fully coupled mitochondria, 0 refers to zero respiratory phosphorylation capacity. Original traces and data are shown in Figures 1-3. The results are expressed as mean±S.E.M. (*N*=4-16).
